# Bactericidal and virucidal action of cetylpyridinium chloride and benzocaine lozenges against common oropharyngeal pathogens

**DOI:** 10.3205/dgkh000530

**Published:** 2025-02-24

**Authors:** Tina Peiter, Fergus de Grey-Warter, Tessa Stahl, Thomas Hallet, Derek Matthews, Maren Eggers

**Affiliations:** 1 Reckitt Benckiser Deutschland GmbH, Heidelberg, Germany; 2 Reckitt Benckiser Healthcare Ltd, Hull, United Kingdom; 3 Reckitt Benckiser Healthcare Ltd, Slough, United Kingdom; 4 Labor Prof. Gisela Enders MVZ GbR, Stuttgart, Germany

**Keywords:** antibacterial agent, virucidal agent, antiseptic agent, drug resistance, benzocaine, cetylpyridinium, lozenges, pharyngitis, bovine coronavirus, influenza virus A

## Abstract

**Aim:**

Too often, antibiotics are prescribed in the treatment of pharyngitis, which can contribute to antimicrobial resistance. We aimed to assess the in vitro antiviral and antimicrobial activity of sugar-free cetylpyridinium chloride (CPC)/benzocaine lozenges, which can potentially offer a more suitable treatment for pharyngitis.

**Methods:**

The antiviral activity of sugar-free CPC/benzocaine (1.4 mg/10 mg) lozenges (Dolo-Dobendan 1.4 mg/10 mg lozenges) was assessed using the DIN EN 14476:2019–10 suspension test against bovine coronavirus (S379 Reims) or influenza virus A (H1N1/Brisbane/59/2007) under clean and dirty conditions. Viral titers were measured after 1, 5, 10, and 30 min exposure; a reduction of ≥4 lg was considered virucidal. For bovine coronavirus, large volume plating was used due to cytotoxicity. Antimicrobial activity was measured against 11 microorganisms associated with pharyngitis, with contact times of 1, 5, and 10 min (+30 min for positive control).

**Results:**

For influenza, sugar-free lozenges showed ≥4 lg efficacy from 5 and 10 min exposure under clean and dirty conditions, respectively. For bovine coronavirus, sugar-free lozenges exhibited ≥4 lg efficacy at 10 min under both conditions. Bactericidal activity was observed against nine of the challenge microorganisms within 5 min, with plate counts of <10 colony-forming units (CFU)/mL for *Pseudomonas aeruginosa*, *Staphylococcus aureus*, *Arcanobacterium haemolyticum*, *Moraxella catarrhalis*, *Porphyromonas gingivalis*, *Prevotelia intermedia*, *Streptococcus dysgalactiae*, and *Streptococcus pyogenes*, and <100 CFU/mL for *Streptococcus pneumoniae. Candida albicans and Escherichia coli* showed counts of <10 CFU/mL at 30 min.

**Conclusions:**

Sugar-free CPC/benzocaine lozenges can be recommended for uncomplicated pharyngitis and may be more appropriate than antibiotics, helping to mitigate antimicrobial resistance.

## Zusammenfassung

**Zielsetzung:**

Bei der Behandlung von Pharyngitis werden Antibiotika zu häufig verschrieben, was zu antimikrobiellen Resistenzen beitragen kann. In der vorliegenden Arbeit wurden die antiviralen und antibakteriellen Eigenschaften von zuckerfreien Lutschtabletten mit Cetylpyridiniumchlorid (CPC) und Benzocain in vitro untersucht. Das stellt möglicherweise eine geeignetere Behandlung für typische Pharyngitiden dar.

**Methode:**

Die antiviralen Eigenschaften von zuckerfreien CPC/Benzocain (1,4 mg/10 mg) Lutschtabletten (Dolo-Dobendan 1,4 mg/10 mg Lutschtabletten) wurden im Suspensionstest nach DIN EN 14476:2019-10 gegen das bovine Coronavirus (S379 Reims) und das Influenzavirus A (H1N1/Brisbane/59/2007) unter hoher und niedriger Belastung untersucht. Die Virustiter wurden nach 1, 5, 10 und 30 min Kontaktzeit gemessen und eine Reduktion von ≥4 lg wurde als viruzid angesehen. Für das bovine Coronavirus wurde zur Reduzierung der Zytotoxizität das Large-Volume-Plating-Verfahren angewendet. Die antimikrobiellen Eigenschaften wurden gegenüber 11 Mikroorganismen, die mit Pharyngitis assoziiert sind, gemessen. Die Kontaktzeiten waren 1, 5 und 10 min (+30 min für die Positivkontrolle).

**Ergebnisse:**

Gegenüber dem Influenzavirus zeigten die Lutschtabletten eine Reduktion von ≥4 lg nach 5 bzw. 10 min sowohl unter niedriger als auch unter hoher Belastung. Die Lutschtabletten zeigten gegenüber dem bovinen Coronavirus unter beiden Bedingungen eine Reduktion von ≥4 lg nach 10 min. Eine bakterizide Wirkung wurde innerhalb von 5 min gegen neun der Mikroorganismen beobachtet, mit <10 koloniebildenden Einheiten (KBE)/mL für *Pseudomonas aeruginosa*, *Staphylococcus aureus*, *Arcanobacterium haemolyticum*, *Moraxella catarrhalis*, *Porphyromonas gingivalis*, *Prevotelia intermedia*, *Streptococcus dysgalactiae* und *Streptococcus pyogenes* und <100 KBE/mL für *Streptococcus pneumoniae. Candida albicans* und *Escherichia coli* wiesen nach 30 min Werte von <10 KBE/mL auf.

**Schlussfolgerung:**

CPC/Benzocain (1,4 mg/10 mg) Lutschtabletten können bei unkomplizierter Pharyngitis empfohlen werden und bieten möglicherweise eine gute Alternative bei nicht zielführender Antibiose und können somit helfen, antimikrobielle Resistenzen einzudämmen.

## Introduction

The cold and flu season occurs mainly during the winter months in temperate climates, and in tropical regions there are irregular outbreaks throughout the year [1], [2]. However, since the COVID-19 pandemic, the timing and duration of flu seasons have become less predictable [3]. Acute pharyngitis (sore throat) is a typical symptom of upper respiratory tract infections (URTIs) and one of the most common reasons for seeking medical care globally [4], [5]. For example, in Germany, pharyngitis accounts for 2.7% of all primary care consultations [6]. 

The most frequent cause of pharyngitis is a viral infection, responsible for around 80% of adult cases [5], [7], [8], [9]. Some of the most common enveloped viruses implicated in the etiology of pharyngitis are coronaviruses, ortho- and paramyxoviruses (e.g., influenza viruses) [10]. A number of bacterial species can also be responsible for pharyngitis, but* Streptococcus (S.) pyogenes* (also known as group A β-hemolytic *Streptococcus* [GABHS]) is the most common bacterial cause of pharyngitis [8], [9]. Antibiotics are prescribed in most cases of pharyngitis, despite the fact that the majority of cases are caused by viral infections, and antibiotics are ineffective against viruses [5], [7], [8], [9]. Most cases of pharyngitis are self-limiting, regardless of the etiology [11], and improve without the need for antibiotics [9]. Inappropriate antibiotic usage for self-limiting conditions, such as pharyngitis, contributes to antimicrobial resistance, which is a global health threat [11], [12]. There is a need for non-antibiotic treatments, such as topical formulations that have antimicrobial and pain-relieving qualities, so that antibiotics can be reserved for bacterial pharyngitis and patients with an increased risk of complications [7], [8]. In general, topical formulations are less likely to cause antimicrobial resistance than systemic antibiotics and have been proven to reliably deliver antiseptically active substances to the throat mucosa [13], [14]. 

Over-the-counter (OTC) medicated throat lozenges containing antiseptics and anesthetics are one option for the symptomatic relief of sore throat. Some have proven efficacy and provide immediate release of active ingredients directly at the site of infection, which offers rapid symptomatic relief [7], [8], [15], [16], [17]. Cetylpyridinium chloride (CPC) is an antiseptic found in throat lozenges that has been described as a successful bactericide against gram-positive and, in higher concentrations, some gram-negative bacteria [10], [17]. CPC also has variable antifungal activity, and some studies suggest its efficacy against certain enveloped viruses [10], [17]. Anesthetics provide symptomatic pain relief and are often combined with antiseptics in throat lozenge formulations [17]. Lozenges containing CPC and benzocaine are well established for the symptomatic relief of sore throat and have a favorable safety profile. These medicated lozenges have been available OTC for several decades in certain countries [10], [18]. However, the virucidal and antimicrobial effects of these lozenges have not been widely reported to date. Using CPC mouthrinse at a concentration of 0.05% results in an immediate reduction in bacterial counts by 2.0 to 2.5 lg steps. The effect reverts to about 1 lg step after 1 hour [19]. CPC is virucidal against both susceptible and resistant strains of influenza viruses at concentrations between 5.0 and 12.5 µg/mL without leading to the development of resistance, and it also reduces influenza-associated mortality and morbidity [20].

As a result of the COVID-19 pandemic, the use of antiseptics considerably increased due to their antiviral properties against SARS-CoV-2 [21]. There is a need for robust antiviral data that are applicable to the clinical situation. The European tiered approach on the virucidal standard, EN 14476, helps provide recommendations regarding the choice of antiseptics that can be used with proven virucidal efficacy [22]. Viral infections are associated with an increased incidence or severity of bacterial co-infections, which may contribute to severe disease [23], [24]. Therefore, combining antiviral and antimicrobial properties in OTC treatments may possibly prevent potential co-infections and improve outcomes. This study assessed the in vitro antimicrobial and antiviral activities of sugar-free CPC/benzocaine lozenges against pathogens known to cause pharyngitis. 

## Materials and methods

### Antiviral method

#### Antiviral test

CPC 1.4 mg/benzocaine 10 mg sugar-free lozenges (Dolo-Dobendan^®^, Reckitt Benckiser Deutschland GmbH, Heidelberg, Germany) were dissolved in 6 mL of distilled water (per lozenge) to make a final concentration of 80%. 

The test sample was prepared according to DIN EN 14476:2019-10 (phase 2/step 1). The sugar-free test samples were added to a suspension of influenza virus A/H1N1/Brisbane/59/2007 or bovine coronavirus strain ‘S379 Riems’ and an interfering substance, which consisted of clean conditions with 0.3 g/L bovine serum albumin (BSA) or a tripartite soil load according to the Organisation for Economic Co-operation and Development (OECD) with 5% BSA fraction V, 5% yeast extract, 0.4% mucosal protein mucin. The test temperature used was 20°C±1°C, which corresponds to the ‘worst case’ test condition according to the EN 14476 standard. The contact times were 1, 5, 10, and 30 min. At the end of each contact time, 0.5 mL of the test sample was immediately diluted in 4.5 mL of an ice-cold maintenance medium to suppress the virucidal action of the test sample. Ten-fold dilutions of the maintenance medium and test sample mixture were transferred onto Madin-Darby canine kidney (MDCK) and *Ovis aries* (CCLV-Rie11) cells in 96-well microtiter plates for influenza and bovine coronavirus, respectively. After incubation at 37°C±1°C in a humidified atmosphere under 5.0% CO_2_, the plates were observed for viral cytopathic effect (CPE), and titers of viral infectivity were calculated using the Spearman and Kärber method (lg 50% tissue culture infectious dose [TCID_50_]/mL with 95% confidence interval). Reduction of virus infectivity was calculated using the differences of lg virus titer with the control (without test lozenge) and after treatment with the lozenge. A reduction of ≥4 lg was considered virucidal. In cases of cytotoxicity in the test samples against bovine coronavirus, a large-volume plating (LVP) method was carried out by inoculating the total volume from the first non-cytotoxic dilution. 

### Antimicrobial method

#### Antimicrobial activity test

CPC 1.4 mg/benzocaine 10 mg sugar-free lozenges (Dolo-Dobendan^®^, Reckitt Benckiser Deutschland GmbH, Heidelberg, Germany) were dissolved at 42–44°C in 5 mL of artificial saliva medium (ARTS), which contains 0.1% meat extract (Sigma 70164), 0.2% yeast extract (LP0021), 0.5% proteose peptone (LP0085), 0.02% potassium chloride (Fisher P/4240), 0.02% sodium chloride (Sigma S7653), 0.03% calcium carbonate (Fisher C/1040), 0.2% glucose (Fisher G/0450), and 0.2% mucin from porcine stomach, type 2 (Sigma M2378), per lozenge. Three test samples were prepared for each challenge microorganism. 

Each CPC/benzocaine test sample (4.9 mL) and positive control (4.9 mL of ARTS) were inoculated with 100 µL of the appropriate challenge microorganisms outlined in Table 1 (Tab. 1), to provide an inoculum level of >1.0x10^4^ colony-forming units (CFU)/mL. The solution was mixed thoroughly using a vortex mixer and incubated at 36–38°C. Inoculum suspensions were prepared in triplicate for each of the 11 challenge microorganisms. Antimicrobial activity was measured after 1-, 5-, 10- and 30-min contact times (30 min only for the positive control) by removing 1 mL of the test sample and placing it into 9 mL of neutralizing diluent with 0.1% peptone water, 1% tween 80, 0.3% lecithin, and sodium chloride (PTLS). Solutions were serially diluted to 10^–5^ and incubated on appropriate nutrient agar medium for at least 3 days (Table 1 (Tab. 1)). Antimicrobial activity (in CFU/mL) was calculated for each challenge microorganism and time point (average of three replicates). 

### Method suitability testing

The test was performed in a manner similar to that of the antimicrobial activity test, with minor differences (Table 2 (Tab. 2)). The method suitability test was carried out to verify that any antimicrobial activity in the sample was effectively neutralized. 

## Results

### Virucidal efficacy

#### Influenza virus

Sugar-free lozenges exhibited a virucidal efficacy of ≥4 lg from 5 and 10 min onwards under clean conditions and with a tripartite soil load, respectively (Table 3 (Tab. 3)). 

#### Bovine coronavirus and cytotoxic effect

Cytotoxicity (deformation of cells and destruction of cell monolayer) made it impossible to achieve the required 4 lg reduction against bovine coronavirus and was detected in the test samples. Therefore, to be able to detect a titer reduction of over 4 lg levels, the LVP method was carried out. Sugar-free lozenges tested against bovine coronavirus showed a virucidal efficacy of 2.67 lg reduction under clean conditions and a 3.4 lg reduction with a tripartite soil load at the 5-min time point. Sufficient virucidal efficacy was also demonstrated at the 10-min time point: ≥3.0 lg reduction under clean conditions and 4.06 lg reduction with a tripartite soil load (Table 4 (Tab. 4)). 

### Microbicidal efficacy

#### Validity of neutralization

For all microorganisms, except *S. pneumoniae*, the counts for the 10^–1^ dilution test result (10^–2^ dilution with *S. pneumoniae*) did not vary by more than a factor of two from the peptone control result. This suggests that any antimicrobial activity present had been sufficiently removed at this dilution and that the method was suitable. The method suitability test was valid because the inoculum levels of each of the microorganism species were 10–150 CFU and there was no growth in any of the negative controls. 

#### In vitro antimicrobial activity of CPC/benzocaine sugar-free lozenges

*S. aureus* and *S. pyogenes* showed counts of <10 CFU/mL at the 5-min time point onwards, whereas *C. albicans* and *E. coli* showed counts of <10 CFU/mL at the 30-min timepoint for all replicates. *S. pneumoniae* showed counts of <100 CFU/mL at the 1-min time point onwards for all replicates. A total of six bacterial species showed counts of <10 CFU/mL at the 1-min time point onwards for all replicates (*A. haemolyticum, M. catarrhalis, P. gingivalis, P. intermedia, P. aeruginosa, S. dysgalactiae*; Table 5 (Tab. 5)). 

## Discussion

OTC antiseptic/anesthetic lozenges are helpful in the management of sore throat, offering easy access to symptomatic relief and providing a more suitable alternative treatment to antibiotics, which contribute to antimicrobial resistance [9], [18]. Most upper respiratory tract infections are self-limiting and do not require antibiotics; therefore, if more patients are encouraged to use self-management options, it could help reduce the inappropriate use of antibiotics [25]. Generating robust in vitro data is essential to understand the efficacy of antiseptics so that they can be presented as a valid alternative to primary-care visits. Antiseptic lozenges are widely available for the treatment of pharyngitis [26], and having a better understanding of their efficacy can support the recommendation of these treatments at the appropriate place in the care pathway, and also differentiate them from non-medicated, purely demulcent throat sweets or moisturizing medicinal devices. CPC/benzocaine is a well-established treatment for pharyngitis [18]; however, it is important to understand its full antimicrobial activity to possibly prevent potential complications and secondary infections. 

This study examined the antiviral and antimicrobial action of sugar-free CPC/benzocaine lozenges. Both the coronavirus and influenza virus are common causes of pharyngitis, and various institutes monitor the infection rates of these viruses [27]. Additionally, the possibility of co-infections with these viruses could impact disease severity [28], [29]. Therefore, these two viruses were included in this study. The findings of this in vitro study confirmed a virucidal efficacy of sugar-free CPC/benzocaine lozenges on the enveloped viruses bovine coronavirus and influenza virus. According to the European standard employed, a reduction of viral infectivity by ≥4 lg levels is regarded as virucidal activity [22]. The sugar-free lozenge demonstrated virucidal activity consistent with the time a lozenge remains in the mouth (within a mean±standard deviation of 6.77±2.01 min) [9]: at 10 min with the bovine coronavirus for both interfering substances; with influenza virus, at 10 min onwards using the tripartite soil load and at 5 min onwards using clean conditions. The results observed for influenza virus H1N1 and bovine coronavirus could be transferable to other influenza viruses and to human variants of coronavirus, respectively [30], [31]. 

This study used bacterial species present in pharyngitis with a broad range of cell structures and sensitivities, including gram-negative anaerobes (*P. intermedia, P. gingivalis*), gram-positive cocci (*S. pyogenes, S. pneumoniae, S. dysgalactiae, S. aureus*), and bacilli *(A. haemolyticum*), as well as gram-negative cocci (*M. catarrhalis*) and bacilli (*P. aeruginosa, E. coli*) [8], [32], [33]. *S. pyogenes* was of particular interest because it is the most frequent cause of bacterial pharyngitis [8], [9]. Additionally, *M. catarrhalis* is often found in conjunction with *S. pyogenes* in patients with certain types of pharyngitis (e.g., tonsillopharyngitis) and has been shown to enhance the adhesion of *S. pyogenes* to the nasopharyngeal epithelium [34], [35], [36]. Although *E. coli* does not typically cause pharyngitis, it was included because of its known connection with antimicrobial resistance [37]. In addition, the study also included *C. albicans*, a yeast that can cause acute pharyngitis [38]. Currently, there are limited data on the efficacy of sugar-free antiseptic/anesthetic lozenges. However, this study showed that sugar-free CPC/benzocaine lozenge demonstrated bactericidal activity against all challenge microorganisms, and results were consistent with the time a lozenge takes to dissolve in the mouth; within 5 min [9], nine of the challenge microorganisms (*P. aeruginosa, S. aureus, A. haemolyticum, M. catarrhalis, P. gingivalis, P. intermedia, S. dysgalactiae, S. pneumoniae*, and *S. pyogenes*) achieved a plate count of <10 CFU/mL. Within 30 min, all of the challenge microorganisms achieved a plate count of <10 CFU/mL. 

Having a lozenge with antiviral and antibacterial properties is important; for instance, influenza virus is activated by furin protease from bacteria, so influenza virus is more infectious if bacteria are present [39]. However, often only antibiotics are prescribed, which does not treat the viral infection [7], [15]. This common misuse can significantly contribute to antimicrobial resistance [7], [12]. Therefore, it is important to use products with both antiviral and antibacterial properties to help avoid unnecessary prescription of antibiotics, and hence reduce antimicrobial resistance [9].

## Limitations

Firstly, only in vitro testing was performed, which does not fully reflect the throat environment and cannot precisely replicate how the lozenges would act in a patient’s throat. For example, the study only focused on planktonic bacteria in isolation; however, the throat may contain a greater range of bacteria. 

In addition, the in vitro method cannot determine the effect of biofilms within the oropharyngeal space, or the effect of the immune system on antimicrobial activity; generating biofilm data and in vivo testing would be an interesting next step. 

However, the in vitro method allows rapid generation of robust data for numerous microorganisms simultaneously. The in vitro/in vivo correlation is a biopredictive mathematical model that can help establish a relationship between in vitro and in vivo efficacy [18]. Therefore, in vitro data could also be used to potentially predict lozenge efficacy in vivo.

## Conclusions

Sugar-free CPC/benzocaine lozenges demonstrated bactericidal activity against all challenge microorganisms, as well as antifungal activity against *C. albicans*, with the possibility of viral load reduction in the throat. Results were consistent with the time a lozenge takes to dissolve in the mouth. Thus, sugar-free CPC/benzocaine lozenges can be recommended as an effective OTC option for patients with uncomplicated pharyngitis, offering rapid antimicrobial and antiviral effects. 

It is important to note that inclusion of the topical anesthetic benzocaine means these lozenges also provide effective pain relief [18], [40]. In contrast, antibiotics do not provide immediate symptomatic relief, nor are they active against the common viral infections, and inappropriate prescription practices will lead to antimicrobial resistance [11]. 

This study provides robust data to demonstrate antiviral, antibacterial and anesthetic effects of CPC/benzocaine lozenges, showing that they are an effective treatment for self-management of self-limiting upper respiratory tract infections. CPC/benzocaine lozenges could also reduce antibiotic prescribing for self-limiting viral pharyngitis and help reduce antimicrobial resistance.

## Notes

### Authors’ ORCIDs

- Tina Peiter: 0009-0009-3056-6180
- Fergus de Grey-Warter: 0009-0005-6494-8161
- Maren Eggers: 0000-0001-8485-9485
- Derek Matthews: 0000-0002-5898-1417

### Funding

This manuscript was provided by Reckitt Benckiser Healthcare International Ltd, UK. 

### Acknowledgments

Medical writing assistance was provided by Isabella Janowski of Elements Communications Ltd., London, UK and was funded by Reckitt Benckiser Healthcare International Ltd, UK. No other sources of support have been involved in the development of the manuscript. 

### Competing interests

Maren Eggers is a paid medical consultant for Reckitt Benckiser Deutschland GmbH, Heidelberg, Germany. Derek Matthews, Thomas Hallett and Fergus de Grey-Warter are employees of Reckitt Benckiser Healthcare Ltd, UK. Tina Peiter and Tessa Stahl are employees of Reckitt Benckiser Deutschland GmbH, Heidelberg, Germany. 

## Figures and Tables

**Table 1:**
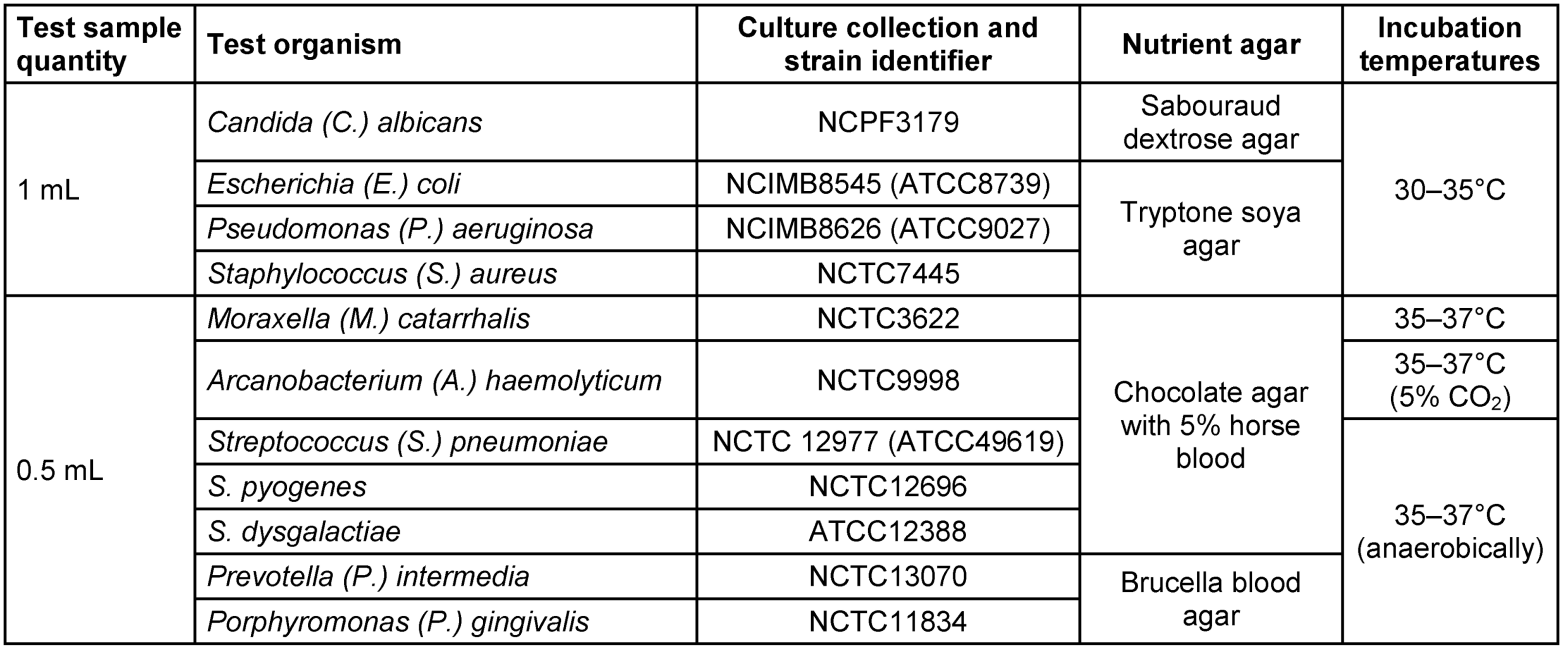
Unique identifier, nutrient agar, and incubation conditions for each test organism (1 mL and 0.5 mL plated)

**Table 2:**
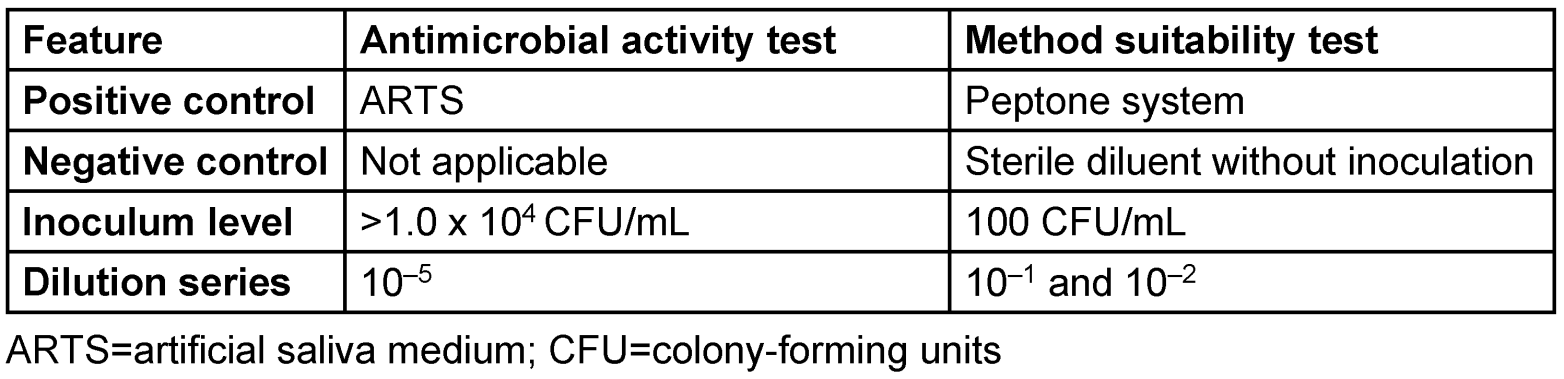
Differences in methods between the method suitability test and the antimicrobial activity test

**Table 3:**
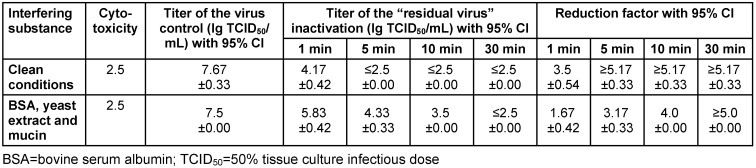
Virucidal effect of CPC/benzocaine sugar-free lozenges against influenza viruses (H1N1)

**Table 4:**
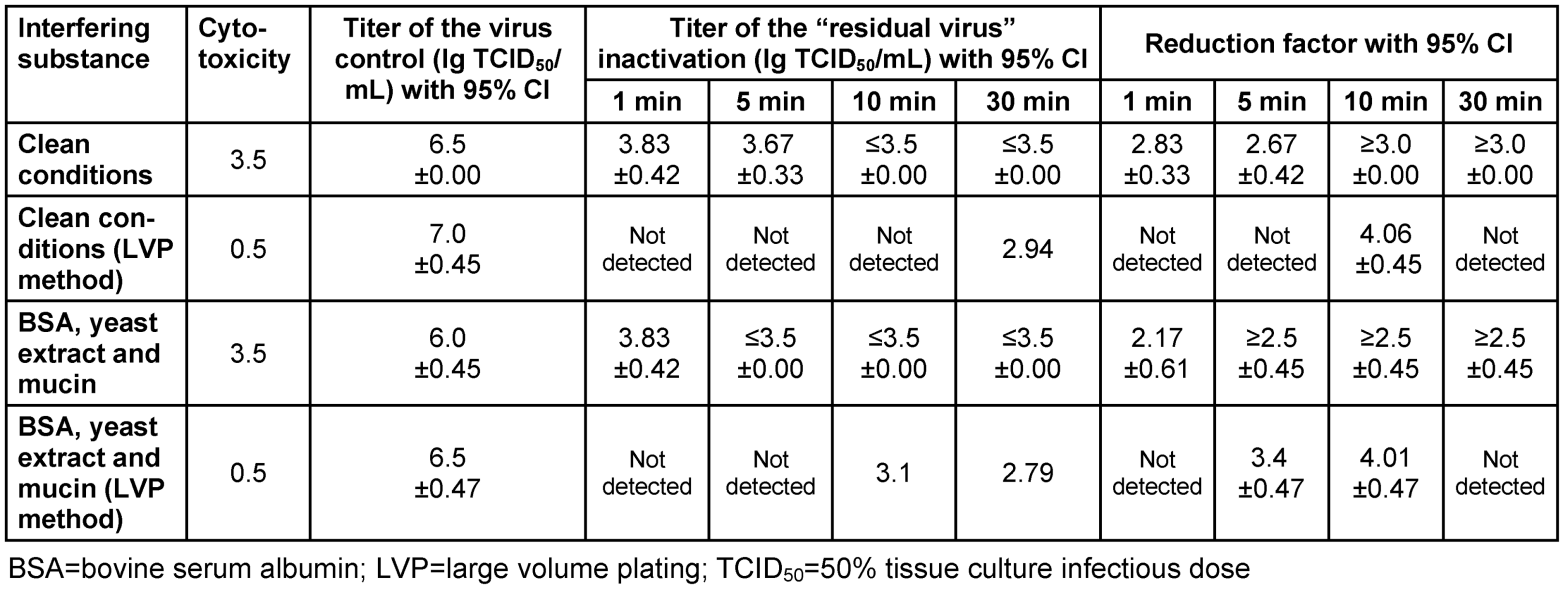
Virucidal effect of CPC/benzocaine sugar-free lozenges against bovine coronavirus

**Table 5:**
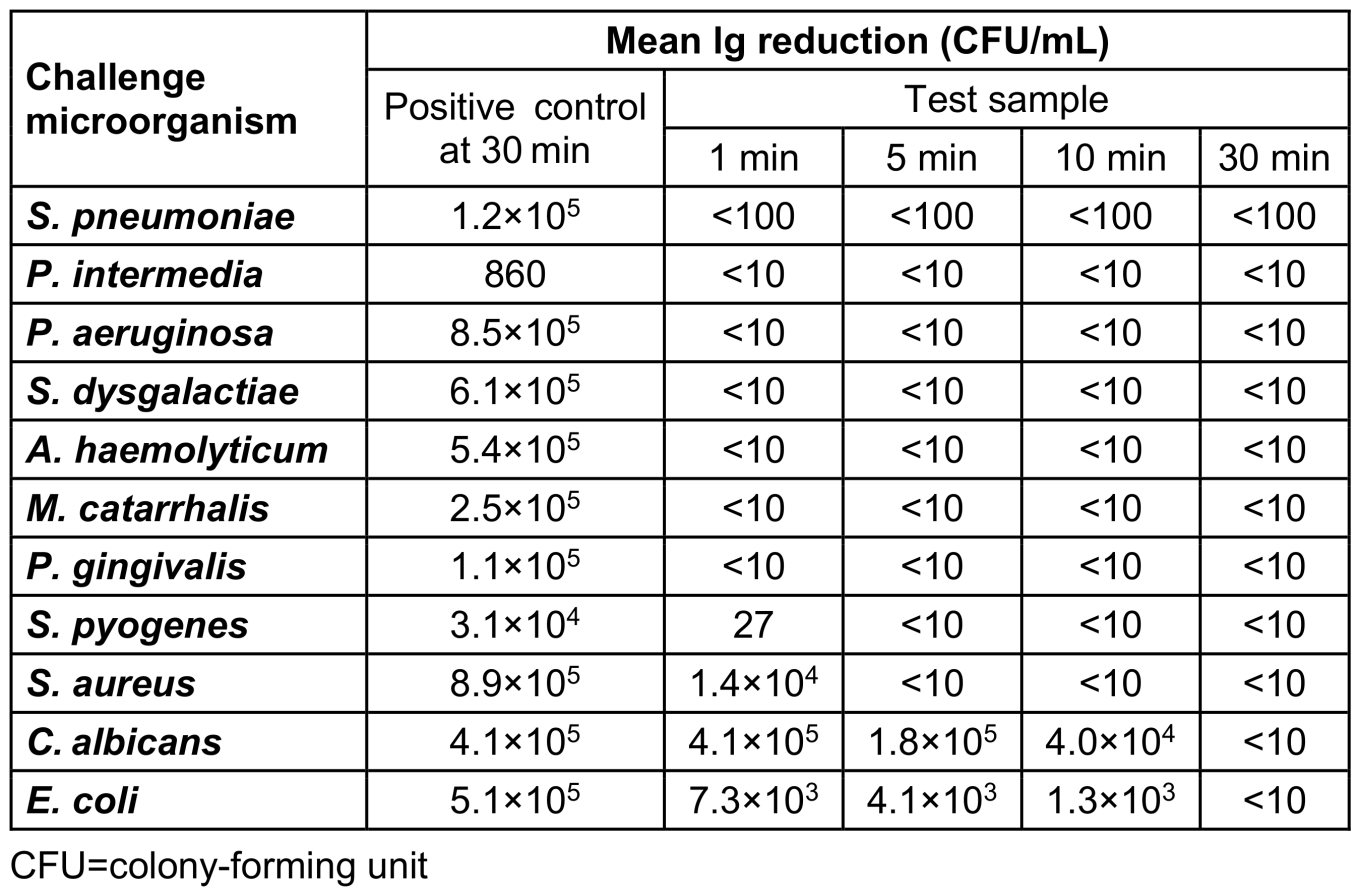
Average colony count of each plate over three replicates for all the challenge microorganisms
